# The *CTGF *-945GC polymorphism is not associated with plasma CTGF and does not predict nephropathy or outcome in type 1 diabetes

**DOI:** 10.1186/1477-5751-10-4

**Published:** 2011-05-08

**Authors:** Amélie Dendooven, Tri Q Nguyen, Lodewijk Brosens, Dongxia Li, Lise Tarnow, Hans-Henrik Parving, Peter Rossing, Roel Goldschmeding

**Affiliations:** 1 Department of Pathology, University Medical Center Utrecht, Heidelberglaan 100, 3584CX Utrecht, The Netherlands; 2 FibroGen Inc, 409 Illinois St. San Francisco, CA 94158 USA; 3 Steno Diabetes Center, A/S Niels Steensens Vej 2, DK-2820 Gentofte, Denmark; 4 Department of Endocrinology, Rigshospitalet University Hospital, Blegdamsvej 9, DK - 2100 Copenhagen, Denmark

## Abstract

The -945GC polymorphism (rs6918698) in the connective tissue growth factor gene promoter (*CTGF/CCN-2*) has been associated with end organ damage in systemic sclerosis. Because CTGF is important in progression of diabetic kidney disease, we investigated whether the -945GC polymorphism is associated with plasma CTGF level and outcome in type 1 diabetes.

The study cohort consisted of 448 diabetic nephropathy patients and 419 normoalbuminuric diabetic patients with complete data concerning renal function and cardiovascular characteristics. Genomic DNA was genotyped by a QPCR-based SNP assay. We observed no relation between the -945GC polymorphism and plasma CTGF level, and the genotype frequencies were not different in nephropathy patients vs. normoalbuminuric controls. General and cardiovascular mortality, and renal function decline was similar in patients with CC, CG or GG genotypes.

In conclusion, the -945GC SNP does not affect plasma CTGF levels, incidence and prognosis of diabetic nephropathy, and cardiovascular outcome.

## Findings

Connective tissue growth factor (CTGF/CCN-2) is a key peptide mediating organ fibrosis [1-3]. Fonseca *et al. *identified a single nucleotide polymorphism (SNP) at position -945 upstream from the transcription initiation site of the *CTGF *gene (-945GC) overrepresented in patients with systemic sclerosis (SSc) and associated with a higher incidence of lung fibrosis [4]. Subsequent studies have either confirmed or questioned the association of the G allele with incidence and severity of SSc, and its relation with *in vivo *CTGF expression levels has not been studied to date [5,6]. Also in diabetic nephropathy, CTGF is an important pathogenic factor, and plasma CTGF levels independently predict mortality and end-stage renal disease (ESRD) [7]. A recent study in hemodialysis patients indicated that the -945GC polymorphism might be associated with cardiovascular, but not all-cause mortality [8]. Therefore, we examined the possible relevance of the -945GC polymorphism for plasma CTGF levels, and for nephropathy and associated manifestations in patients with type 1 diabetes.

General characteristics and baseline parameters of patients are summarized in Table 1.

**Table 1:** Patient characteristics at baseline

	Diabetic Nephropathy	Normoalbuminuria	P-value
**Patient characteristics**			

N (% male)	448 (60.9)	419 (54.4)	p = 0.02

Age (years)	42.2 ± 10.5	45.3 ± 11.5	p < 0.001

Duration of DM (years)	28.2 ± 8.7	27.8 ± 10.1	p = 0.025

BMI (kg/m^2^)	24.2 ± 3.3	24.1 ± 2.9	p = 0.7

Retinopathy (nil/simplex/proliferative)	7/135/306	151/159/109	p < 0.001

Antihypertensiva (no/yes)	95/308	351/68	p < 0.001

Smokers (%)	46	39	p = 0.05

			

**Glycemic control**			

Blood glucose (mmol/l)	11.0 ± 5.4	9.4 ± 4.7	p < 0.001

HbA1c (%)	9.4 ± 1.5	8.4 ± 1.1	p < 0.001

			

**Parameters of Nephropathy**			

UAE (mg/24 h)	593.1 (250.0-1519.5)	7.0 (4.0-12.0)	p < 0.001

Plasma creatinine (μmol/l)	102 (82.0-136.3)	79 (53-81)	p < 0.001

GFR (ml/min/1.73 m^2^)	66.1 ± 27.7	87.4 ± 14.9	p < 0.001

ESRD (%male)	24 (70.8)	0	p < 0.001

Systolic Blood Pressure (mm Hg)	144.3 ± 21.8	133.8 ± 18.6	p < 0.001

Diastolic Blood Pressure (mm Hg)	82.5 ± 12.2	76.1 ± 9.6	p < 0.001

Data are presented as mean ± SD, median (interquartile range), or N (%). The study was performed according to the principles of the Declaration of Helsinki and approved by the ethical committee of Copenhagen County. All patients gave informed consent.

Smoking and body mass index (BMI) did not differ significantly between diabetic nephropathy and normoalbuminuric subjects. Retinopathy, blood pressure, use of antihypertensive medication, and parameters of nephropathy were all higher in the diabetic nephropathy group as compared to the normoalbuminuric subjects.

Genomic DNA was genotyped by a Custom-Taqman-SNP-Genotyping-Assay (Applied Biosystems, Foster City, CA, USA) for the GC polymorphism at position -945. The distributions of the genotypes were in accordance with the Hardy-Weinberg equilibrium for the entire population (p = 0.52), and the subgroups divided by presence or absence of nephropathy (p = 0.49 and p = 0.10 respectively). Genotype frequencies were very similar between diabetic nephropathy and diabetic normoalbuminuric patients, with a frequency of the G allele of 22.8% in the DN group as compared to 21.9% in the NA group (p = 0.481) (Table 2). The power of the study was determined using web-based software (http://www.stat.ubc.ca/~rollin/stats/ssize/b2.html). This showed a power of 95% for detection of a 10% increase in DN patients of the GG genotype frequency, i.e. an increase to 31.9% in DN as compared to the 21.9% in the NA patients which was comparable with the previously observed range of 30 to 20% in diseased vs. control groups [4].

**Table 2:** Distribution of genotype and allele frequencies for the *CTGF *promoter polymorphism at -945

	Genotype frequencies (%)		Total	P-value
		
*CTGF *-945GC polymorphism	Diabetic Nephropathy	Normoalbuminuria		
CC	126 (28.1)	126 (30.1)	252 (29.0)	0.481
	
CG	220 (49.1)	201 (48.0)	421 (48.6)	
	
GG	102 (22.8)	92 (21.9)	194 (22.4)	
	
**Total**	448 (100)	448 (100)	867 (100)	

	**Allele frequencies (%)**		**Odds ratio**	**P-value**
		
**CTGF -945 promoter polymorphism**	**Diabetic Nephropathy**	**Normoalbuminuria**		

Allele C	472 (52.7)	453 (54.0)	1.057	0.596
		
Allele G	424 (47.3)	385 (46.0)		

P-values were calculated using Fisher's exact test and Chi-square analysis respectively. There is no difference in genotype or allele frequencies between diabetic nephropathy patients and normoalbuminuric patients at baseline.

**Figure 1:**
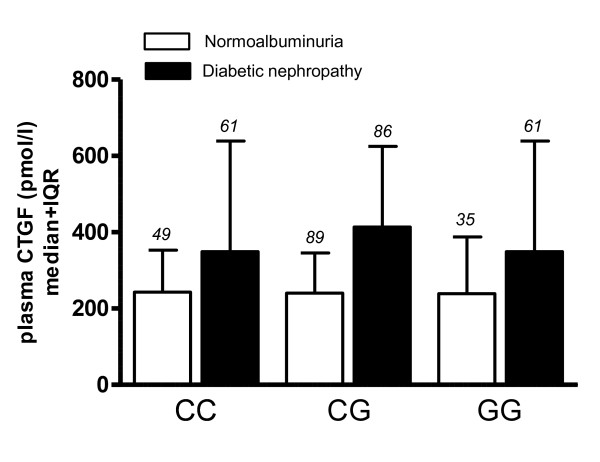
**Relation of plasma CTGF levels (pmol/l) with genotype**. Bars are median+interquartile range. White bars: normoalbuminuric diabetic patients (N = 173); black bars: diabetic patients with nephropathy (N = 198). Plasma CTGF levels are higher in DN (p < 0.0001, ANOVA on log transformed values for conversion to a normal distribution of positively skewed data). There is no significant difference in plasma levels according to genotype. Number of patients in each group is indicated in italics above the error bars.

Plasma CTGF levels were determined in a subset of 381 by a sandwich enzyme-linked immunosorbent assay (ELISA) using monoclonal antibodies against two distinct epitopes of human CTGF (FibroGen, San Francisco, CA) as described previously [7]. Diabetic nephropathy was associated with significantly elevated CTGF levels (381.3 pmol/l (270.3-626.4) in DN vs. 235.2 pmol/l (168.1-352.9) in normoalbuminuria, p < 0.0001). However, there was no difference in CTGF levels between genotypes (Figure 1). Also, linear regression analysis could not predict plasma CTGF levels from genotype (not shown).

The mean follow-up time in the diabetic nephropathy group was comparable to that in the normoalbuminuric group, 9.0 ± 3.3 and 8.6 ± 3.2 years, respectively. At follow-up, the presence of renal and cardiovascular endpoints was compared with genotype. There were no significant differences between the CC, CG and GG genotypes in terms of mortality or development of ESRD in the total population under study (Table 3). Also, in separate analyses of normoalbuminuric patients and patients with nephropathy there was no difference in incidence of ESRD, total mortality, cardiovascular mortality, or non-fatal cardiovascular events between the different genotypes (all p > 0.05). None of the normoalbuminuric patients developed ESRD over the studied period.

**Table 3:** Association of the *CTGF *promoter polymorphism at -945 with clinical outcomes at follow-up

			CC (%)	CG (%)	GG (%)	Total (%)	P-value
**Diabetic nephropathy**	**Mortality**	Yes	42 (30)	62 (44)	36 (26)	140 (100)	0.369
			
		No	84 (27)	158 (51)	66 (22)	308 (100)	
	
	**Cardiovascular (CV) death**	Yes	20 (27)	35 (48)	18 (25)	73 (100)	0.915
			
		No	106 (28)	185 (49)	84 (23)	375 (100)	
	
	**Non-fatal CV event**	Yes	40 (35)	50 (43)	25 (22)	115 (100)	0.173
			
		No	86 (26)	170 (51)	77 (23)	333 (100)	
	
	**End-stage renal failure**	Yes	29 (30)	46 (47)	23 (23)	98 (100)	0.886
			
		No	97 (28)	174 (50)	79 (22)	350 (100)	


**Normoalbuminuria**	**Mortality**	Yes	14 (40)	15 (43)	6 (17)	35 (100)	0.394
			
		No	112 (29)	186 (48)	86 (22)	384 (100)	
	
	**Cardiovascular (CV) death**	Yes	4 (31)	6 (46)	3 (23)	13 (100)	0.991
			
		No	122 (30)	195 (48)	89 (22)	406 (100)	
	
	**Non-fatal CV event**	Yes	15 (37)	20 (49)	6 (14)	41 (100)	0.415
			
		No	111 (29)	181 (48)	86 (23)	378 (100)	
	
	**Microalbuminuria**	Yes	21 (38)	25 (45)	9 (16)	55 (100)	0.306
			
		No	105 (29)	176 (48)	83 (23)	364 (100)	
	
	**Development of nephropathy**	Yes	0 (0)	1 (100)	0 (0)	1 (100)	0.581
			
		No	126 (30)	200 (48)	92 (22)	418 (100)	

There is no effect of the -945GG genotype on either mortality, fatal or non-fatal cardiovascular events, or development of renal disease. P-values are calculated using Fisher's exact test.

Altogether, this makes it doubtful that the -945GC polymorphism plays a major role in susceptibility to developing DN. Apparently, the association of the *CTGF *-945GC SNP with disease is not the same in all patient groups and categories, as has been noted in previous studies that could not always confirm the originally observed association of the -945GC SNP with Ssc [4,5]. Although, theoretically, population differences might affect the apparent contribution of SNPs to disease manifestations, one of these reports examined a large number of patients of diverse nationality and ethnicity but could not replicate the association of the G allele with SSc [6].

It has been observed that CTGF levels are higher in Ssc patients as compared to healthy controls [9], but a possible association of serum or plasma CTGF levels with genotype has not been assessed. This hampers an adequate interpretation of the effect of the polymorphism on *in vivo CTGF *transcription and translation. Therefore, we compared genotype differences for the -945CG polymorphism with plasma CTGF levels in DN and NA patients with diabetes. We found that plasma CTGF levels were not associated with this polymorphism, which further questions its relevance in diabetic kidney disease. In contrast, it has recently been shown that the G allele of an SNP (with a population frequency of around 5%) at -20 in the promoter region of the *CTGF *gene was associated with an increased risk towards developing micro- and macroalbuminuria via increased *CTGF *promoter activity depending on Smad1 [10]. It will be interesting to learn whether this SNP affects plasma CTGF levels.

Studies regarding other SNPs in the *CTGF *promoter have been published before, and most of these deny a contribution of CTGF SNPs to human disease. Three other potentially functional SNPs in the *CTGF *gene (at positions -650, -484 and 247) have been reported not to be associated with diabetic nephropathy [11]. A large study using transmission equilibrium testing revealed no relationship with diabetic nephropathy at yet another SNP (rs9493150) in the *CTGF *gene [12]. In a study from Thailand, an SNP at position -447 was analysed in the context of biliary atresia and no association was observed with either incidence of biliary atresia or occurrence of postoperative jaundice [13]. Finally, none of six *CTGF *gene polymorphisms (including the -945GC SNP) studied in chronic hepatitis C infection was associated with the severity of hepatic fibrosis [14]. However, a recent study in a French population did show that the frequency of the rs9399005TT genotype was lower in Ssc than in control patients, and that the T allele was associated with altered mRNA stability [15]. This is an interesting finding awaiting validation in independent studies of Ssc patients, given the large discrepancies between different studies on polymorphisms even in the same disease. To conclude, in our cohort of 867 Northern European type 1 diabetes patients, the previously described -945GC SNP appears not to have a major impact on plasma CTGF levels, incidence and prognosis of nephropathy, and cardiovascular outcome.

## List of abbreviations

ANOVA: analysis of variance; BMI: body mass index; CTGF: connective tissue growth factor; DM: diabetes mellitus; DN: diabetic nephropathy; ELISA: enzyme-linked immunosorbent assay; ESRD: end-stage renal disease; GFR: glomerular filtration rate; NA: normoalbuminuria; NS: non significant; QPCR: quantitative PCR; SD: standard deviation; SNP: single nucleotide polymorphism; SSc: systemic sclerosis; UAE: urinary albumin excretion.

## Competing interests

Roel Goldschmeding has been employed by and received research support from FibroGen Inc., San Francisco, CA. Dongxia Li is currently employed by the same institution. The other authors have nothing to declare.

## Authors' contributions

AD carried out the genotyping assays, analyzed the data and wrote the manuscript. TQN participated in the design of the study and helped revise the manuscript. LB helped set up the genotyping assay and helped revise the manuscript. DL validated the CTGF ELISA assay. HHP, LT and PR set-up the patient database and provided DNA and plasma samples, PR and LT also helped revise the manuscript. RG conceived of the study, supervised its design and coordination and revised the manuscript. All authors read and approved the manuscript.
